# Comparative Molecular Evolution of *Trichoderma* Chitinases in Response to Mycoparasitic Interactions

**DOI:** 10.4137/ebo.s4198

**Published:** 2010-03-15

**Authors:** Katarina Ihrmark, Nashwan Asmail, Wimal Ubhayasekera, Petter Melin, Jan Stenlid, Magnus Karlsson

**Affiliations:** 1 Department of Forest Mycology and Pathology, Swedish University of Agricultural Sciences, Box 7026, S-75007, Uppsala, Sweden; 2 Department of Molecular Biology, Swedish University of Agricultural Sciences, Biomedical Center, Box 590, S-75124, Uppsala, Sweden; 3 Department of Microbiology, Swedish University of Agricultural Sciences, Box 7025, S-75007, Uppsala, Sweden; 4 MAX-lab, Lund University, Box 118, S-221 00 Lund, Sweden and Institute of Medicinal Chemistry, University of Copenhagen, Universitetsparken 2, DK-2100 Copenhagen Ø, Denmark. Email: magnus.karlsson@mykopat.slu.se

**Keywords:** protein evolution, *Trichoderma*, mycoparasitism, chitinase

## Abstract

Certain species of the fungal genus *Trichoderma* are potent mycoparasites and are used for biological control of fungal diseases on agricultural crops. In *Trichoderma*, whole-genome sequencing reveal between 20 and 36 different genes encoding chitinases, hydrolytic enzymes that are involved in the mycoparasitic attack. Sequences of *Trichoderma* chitinase genes *chi18-5*, *chi18-13*, *chi18-15* and *chi18-17*, which all exhibit specific expression during mycoparasitism-related conditions, were determined from up to 13 different taxa and studied with regard to their evolutionary patterns. Two of them, *chi18-13* and *chi18-17*, are members of the B1/B2 chitinase subgroup that have expanded significantly in paralog number in mycoparasitic *Hypocrea atroviridis* and *H. virens. Chi18-13* contains two codons that evolve under positive selection and seven groups of co-evolving sites. *Chi18-15* displays a unique codon-usage and contains five codons that evolve under positive selection and three groups of co-evolving sites. Regions of high amino acid variability are preferentially localized to substrate- or product side of the catalytic clefts. Differences in amino acid diversity/conservation patterns between different *Trichoderma* clades are observed. These observations show that *Trichoderma* chitinases *chi18-13* and *chi18-15* evolve in a manner consistent with rapid co-evolutionary interactions and identifies putative target regions involved in determining substrate-specificity.

## Introduction

Fungi are predominant pathogens on plants and infections have traditionally been controlled by chemical fungicides. Concerns about environmental impact of fungicides have made biological control an attractive option for managing plant diseases. Several mycoparasitic species of the anamorphic fungal genus *Trichoderma* are currently being used as biocontrol agents, e.g. *Trichoderma harzianum* (teleomorph *Hypocrea lixii*), *T. virens* (teleomorph *H. virens*), *T. atroviride* (teleomorph *H. atroviridis*) and *T. asperellum*.^1^ For simplicity, we refer to *Trichoderma* and *Hypocrea* as *Trichoderma* in this study. *Trichoderma* spp. are frequently isolated from temperate and tropical soils, where they colonize woody and herbaceous material. Several mechanisms are proposed to be involved in the biocontrol ability of *Trichoderma* species, including direct mycoparasitic attack on plant pathogenic species, competition for plant exudates, nutrients or space, induced local and systemic response, and enhancement of plant growth.^2^ The mycoparasitic attack often includes sensing and directed growth towards the antagonist,^3^ followed by attachment and formation of appressoria. *Trichoderma* then secrete several cell wall degrading enzymes and mycotoxic peptaibol metabolites.^4^,^5^ Chitin is an important constituent of fungal cell walls and chitinases have been shown to contribute to mycoparasitic attack.^6^

Fungal chitinases (EC3.2.1.14) exclusively belong to family 18 glycoside hydrolases and they are all predicted to possess a retaining mode of action.^7^ Chitinases are involved in different biological functions such as cell wall remodelling during growth and development, degradation of chitin for nutritional needs and aggressive interactions with other fungi, insects and nematodes.^8^,^9^ Whole genome sequencing of three different *Trichoderma* species, *H. jecorina*, *H. atroviridis* and *H. virens*, has revealed that a large number of chitinase genes are present in these species (20, 29 and 36 genes respectively).^10^,^11^ Orthologs to the following chitinase genes have been previously cloned and studied from various *Trichoderma* species: *chi18-2*, *chi18-3*, *chi18-4*, *chi18-5*, *chi18-6*, *chi18-7*, *chi18-10*, *chi18-12*, *chi18-13*, *chi18-15* and *chi18-17*.^10^ Based on previous work on phylogenetic relationships between chitinase catalytic domains, chitinases are divided into three main groups, A, B and C. These groups are further subdivided into subgroups A2-A5, B1-B5 and C1-C2.^7^ In certain *Trichoderma* chitinases, the catalytic domain is connected to substrate-binding domains which are not necessary for chitinolytic activity, but may enhance the efficiency of the enzymes.^12^,^13^ Transcriptional patterns of *Trichoderma* chitinases show that some are expressed in response to mycoparasitic conditions, orthologs to *chi18-5*, *chi18-7*, *chi18-10*, *chi18-12*, *chi18-13*, *chi18-15* and *chi18-17*,^10^,^14^–^18^ while others are constitutively expressed, *chi18-2*, *chi18-3* and *chi18-4*.^10^

Chitinase groups B and C are reported to display gene copy number expansions in some soil-borne ascomycetes, in addition to low levels of inter- and intraspecific amino acid conservation, which can be interpreted as a result of diversifying selection.^7^ *Chi18-15* has been previously shown to be of actinobacterial origin and horizontally transferred to *Trichoderma*.^12^ In addition, some plant defence chitinases from the genus *Arabis* and the family *Poaceae* have evolved rapidly in response to a co-evolutionary arms race between plant host and fungal pathogen, resulting in a continuous selection for adaptive modifications.^19^,^20^

We hypothesize that *Trichoderma* chitinases, which have a function during the mycoparasitic interaction, have evolved adaptations to specific ecological contexts, such as cell wall composition of hosts, antagonistic microbial chitinase inhibitors and other environmental factors, of different *Trichoderma* species. We assume that specificity-determining residues have undergone mutations to compensate for the specificity needed; hence in paralogous or closely related orthologous sequences, specificity-determining residues may tend to display greater diversity than other positions. This concept was tested on four different *Trichoderma* chitinases that have been implicated in having a function during the mycoparasitic attack, *chi18-5*, *chi18-13*, *chi18-15* and *chi18-17*, by analyzing distribution of amino acid diversity, evolutionary rates and presence of co-evolving codons. In addition, we analyzed chitinase gene copy number expansions as the mycoparasitic lifestyle may have promoted the conservation of new paralogs.

We detected distinct differences in evolutionary trajectories that identify *chi18-13* and *chi18-15* as likely targets for adaptive evolution during mycoparasitic interactions. The results suggest that fungal-fungal interactions can drive adaptive changes in enzymatic properties as a response to specific ecological contexts of different *Trichoderma* species.

## Materials and Methods

### Fungal material and media

Fifteen different strains of *Trichoderma* were used in the study (Table 1). Species assignment was based on analyses of species specific oligonucleotide barcodes located within the internal transcribed spacers 1 and 2 (ITS1 and ITS2) regions of the rRNA repeat, amplified by primers ITS1F and ITS4,^21^,^22^ by using *Trich*OKey version 2.^23^ Strains were maintained on malt extract agar at 25 °C.

### DNA extraction

For DNA extraction, conidia were transferred to liquid media (2% wv^−1^ glucose, 2% wv^−1^ yeast extract, 1% wv^−1^ peptone) and incubated on a shaker for 16 to 54 hours. Mycelia were harvested, freeze-dried overnight, and crushed with a toothpick. An equal volume of DNA extraction buffer (0.2 M Tris (pH 8.5), 0.25 M NaCl, 0.5% SDS, 25 mM EDTA) was added and the mixes were incubated for 30 min at 70 °C followed by micro-centrifugation at maximum speed for 5 minutes. Supernatants were RNase treated, extracted by phenol and chloroform followed by isopropanol precipitation. DNA pellets were dissolved and adjusted to 100 ng μl^−1^ in 10 mM Tris (pH 8).

### Primer design and PCR

The coding regions of four chitinases, *chi18-5*, *chi18-13*, *chi18-15* and *chi18-17* were amplified from at least one strain per species, using primers listed in Supplemental Table S1. Sequences from the *H. jecorina*, *H. atroviridis* and *H. virens* genome projects (http://www.jgi.doe.gov/) were used for initial primer design; by aligning protein ID 80833 from *H. jecorina* and 111866 *H. virens* (*chi18-5*), 119859 *H. jecorina*, 45585 *H. atroviridis* and 25421 *H. virens* (*chi18-13*), 59791 *H. jecorina* and 89999 *H. virens* (*chi18-15*) and 110317 *H. jecorina* and 42107 *H. virens* (*chi18-17*) in the program BioEdit.^24^ Primers were designed in conserved regions and evaluated with the program Primer3.^25^ PCR was performed using 2720 and Veriti thermal cyclers (Applied Biosystems). The amplification was run with approximately 0.5 ng μl^−1^ template DNA in a solution of 0.2 mM dNTP-mix, 0.03 U μl^−1^ ThermoRed Taq DNA polymerase with buffer Y and enhancer P according to the manufacturer’s instruction (Saveen and Werner) and 0.2–0.6 μM of each primer (the more degenerate the primer the higher the primer concentration) and 2.75 mM MgCl_2_. An initial denaturation step at 94 °C for 5 min was followed by 35 amplification cycles of denaturation at 94 °C for 30 s, annealing at 45–60 °C for 30 s and extension at 72 °C for 30–90 s. The thermal cycling was ended by a final extension step at 72 °C for 7 min. The PCR products were separated by gel electrophoresis on 1% agarose gels, purified with AMPure (Agencourt) and sequenced with a CEQ 8000 with the GenomeLab DTCS Quick Start Kit (Beckman Coulter). Sequence analyses and alignments were performed with the DNASTAR program package (DNAstar). Sequences were deposited in GenBank with accession numbers GU180607, GU186414-GU186439.

### Likelihood analysis of gene gain and loss

Using a species phylogeny and chitinase gene copy number in extant species as input data (Supplemental Fig. S1), the program CAFE (Computational Analysis of gene Family Evolution) was used to test for accelerated rates of chitinase family expansions or contractions and identification of branches responsible for the non-random evolution.^26^,^27^ Total number of chitinase genes for *H. atroviridis* was 29 (7 group A, 13 group B and 9 group C) and for *H. virens* 36 (8 group A, 13 group B and 15 group C).^11^ In the species phylogeny (Fig. S1), *H. atroviridis* and *H. virens* were set as sister taxa, with a divergence time of 180 million years. They were in turn separated from *H. jecorina* by 10 million years. All additional species and divergence dates were as described previously.^7^ The birth and death parameter (λ) was estimated from the data and was 0.001 for all datasets. *P*-values were computed using 1000 re-samplings. Identification of the branch that was the most likely cause of deviations from a random model was determined by Viterbi and Likelihood ratio test procedures.^26^ We considered *P*-values ≤ 0.05 or likelihood ratios above 50 to be significant for branch identification.

### Phylogenetic analysis

Translated gene products from the *H. atroviridis* and *H. virens* genome sequences were screened for the presence of chitinases using an iterative BLAST approach.^7^,^28^ Amino acid sequences of chitinase catalytic domains were determined using InterProScan.^29^ Sequences were aligned with Clustal W implemented in MEGA 4.0.2 using default settings and trimmed manually.^30^,^31^ Phylogenetic analyses were performed using Neighbour-joining implemented in MEGA 4.0.2,^31^ using either complete or pair wise deletion of gaps and missing data, and either a Poisson correction or the JTT substitution model.^32^ Statistical support for phylogenetic grouping was assessed by 1000 bootstrap resamplings.

### Reverse conservation analysis (RCA)

From amino acid alignments of chitinases we identified regions of low conservation between closely related orthologs by applying RCA. Introns were removed from DNA sequences before translation. Amino acid sequences were aligned by Clustal X,^33^ and RCA analyses were performed as described by Lee (2008).^34^ In short, Rate4Site (Version 2.01) was used to calculate the degree of conservation (S score, high scores correspond to low degree of conservation) for each amino acid position using the empirical Bayesian method.^35^,^36^ However, the S score varied considerably from residue to residue and was difficult to analyze visually. Therefore, a sliding-window average (n = 7) of normalized S scores (mean was 0 and standard deviation was 1) was plotted in Excel (Microsoft) (W mean score) and significant peaks were defined by intensity (I) values of 0.5 (i.e. 0.5 standard deviation), as recommended by Lee (2008).^34^

### Analysis of codon-usage

Differences in codon-usage between fungal and bacterial chitinases was analysed by performing multivariate (correspondence) analysis using the program CodonW,^37^ accessed through the Mobyle web interphase (http://mobyle.pasteur.fr/cgi-bin/portal.py?form=codonw). In-frame, protein-encoding DNA sequences were used as input data. The universal genetic code was used, correspondence analysis was performed on codon usage frequencies, and all other options were set to default. Differences in codon-usage between genes were visualized by plotting the position of each gene on the resulting coa-axis 1 and 2 in Excel (Microsoft).

### Homology modelling of chitinases

Similar sequences were located in the protein entries of GenBank,^38^ and aligned using Clustal W and hidden Markov models.^30^,^39^ Family 18 chitinase catalytic domain structures were obtained from the Protein Data Bank (PDB),^40^ then superimposed and compared with the programs LSQMAN and O.^41^,^42^ Multiple sequence alignments were used to generate the best pair wise alignments, which were the basis for generating homology models of the catalytic modules of *H. jecorina* chitinases in the program SOD.^41^ *Aspergillus fumigatus* chitinase (PDB entry 1W9P identity 60%),^43^ was used as a template for modelling chi18-5, Chimerolectin from *Parkia platycephala* seeds (PDB entry 2GSJ.pdb identity 43%),^44^ was used for chi18-13, *Streptomyces coelicolor* chitinase (PDB entry 3EBV.pdb (unpublished) identity 38%) was used for chi18-15 and *Saccharomyces cerevisiae* chitinase 1 (PDB entry 2UY2.pdb identity 47%),^45^ was used for chi18-17. The models were adjusted in O, using rotamers that would improve packing in the interior of the protein and accounting for insertions and deletions in loop regions. The models are available upon request from the authors. The figure was prepared using MOLSCRIPT and Molray.^46^,^47^

### Codon-based analyses

Percent nucleotide identity between pair wise comparisons of chitinase genes were performed in MegAlign, implemented in the DNASTAR program package (DNASTAR).

The rate of non-synonymous (dN) and synonymous (dS) substitutions at each codon, and identification of sites evolving under positive or negative selection, was determined using the random effects maximum likelihood models (REL),^48^ implemented in the HyPhy software package,^49^ accessed through the Datamonkey webserver.^50^ As recommended when using REL, the optimal nucleotide substitution model was estimated for each gene separately,^48^,^51^ and included the following modifications to the general reversible nucleotide model;^52^–^54^ *chi18-13*: C↔T: R_CT_ and *chi18-15*: A↔C: R_AC_, A↔T: R_AC_, C↔G: R_CG_, C↔T: R_CT_, G↔T: R_CG_. A Bayes factor value ≥50 (default) was used as an indication of strong positive selection at a site, while values between 10 and 49 were considered to indicate weak support of positive selection.^48^

Identification of co-evolving sites was done using the Spidermonkey/BGM program implemented in the HyPhy software package,^49^,^55^ accessed through the Datamonkey webserver.^50^ The same nucleotide substitution models as were used for REL analysis were used. Global dN/dS values were estimated by the program, ambiguous characters were averaged, a two-parent, directed network was used and sites were selected based on non-synonymous branch counts (threshold ≥3). A posterior probability value ≥0.5 (default) was used as a definition of association between sites.

## Results

### Likelihood analysis of gene gain and loss

The size of the fungal chitinase gene family, including *H. jecorina*, *H. atroviridis* and *H. virens*, was tested for compatibility with a stochastic birth and death model using the program CAFE.^26^,^27^ Previous results show that cluster A chitinases are closely related with cluster C and in order to assign expansions to chitinase subgroups, the data was analysed in three ways; cluster A + C chitinases separately, cluster B chitinase separately and all chitinases merged. The analyses showed that the fungal chitinase gene family, analysing groups A + C and group B separately, as well as all chitinases merged, have evolved nonrandomly (*P* ≤ 0.015) (Fig. S1). When analysing all chitinases merged, the branches for both *H. atroviridis* and *H. virens* were identified as contributing to the non-random pattern (*P* ≤ 0.006), compared with only the *H. virens* branch when groups A + C was analysed separately (*P* < 0.001). The analysis of group B chitinases separately identified a non-random pattern for branches leading to *H. atroviridis*, *H. virens* and the ancestor to the *Trichoderma* clade as well as the ancestor to *H. atroviridis*/*H. virens* (*P* ≤ 0.035).

Analysis of gene phylogenies of chitinase subgroups identified subgroups C1 and C2 as the likely targets for the observed non-random gene copy number expansion in *H. virens* (Supplemental Fig. S2), as compared with other Sordariomycetes. *H. atroviridis* also contained a high number of C group chitinase genes, although the expansion was not statistically significant in the current analysis. Another observation was that while *H. virens* contained high numbers of both C1 and C2 chitinases, *H. atroviridis* contained mainly C1 chitinases and *H. jecorina* contained exclusively C2 members (Fig. S2). A more detailed analysis of group B chitinases revealed that the non-random expansion in the *Trichoderma* clade took place in the B1/B2 subgroup cluster (Fig. S2).

### Reverse conservation analysis of chi18-5 amino acid variability

**Figure 1.**
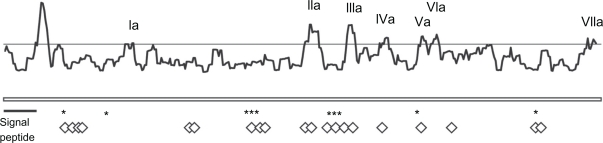
Reverse conservation analysis of chi18-5 orthologs. Amino acid diversity was estimated using Rate4Site, based on a Clustal X alignment of chi18-5 *Trichoderma* orthologs, and plotted as W mean scores. The y-axis represents arbitrary units (not shown) while a horizontal line indicates a 0.5 standard deviation cutoff. The x-axis represents residue position, asterisks (*) indicate positions of catalytic residues, diamonds (⋄) indicate substrate-interacting residues. The positions of the signal peptide and regions with high amino acid diversity successfully visualised by homology modelling are indicated (Ia–VIIa).

**Figure 2.**
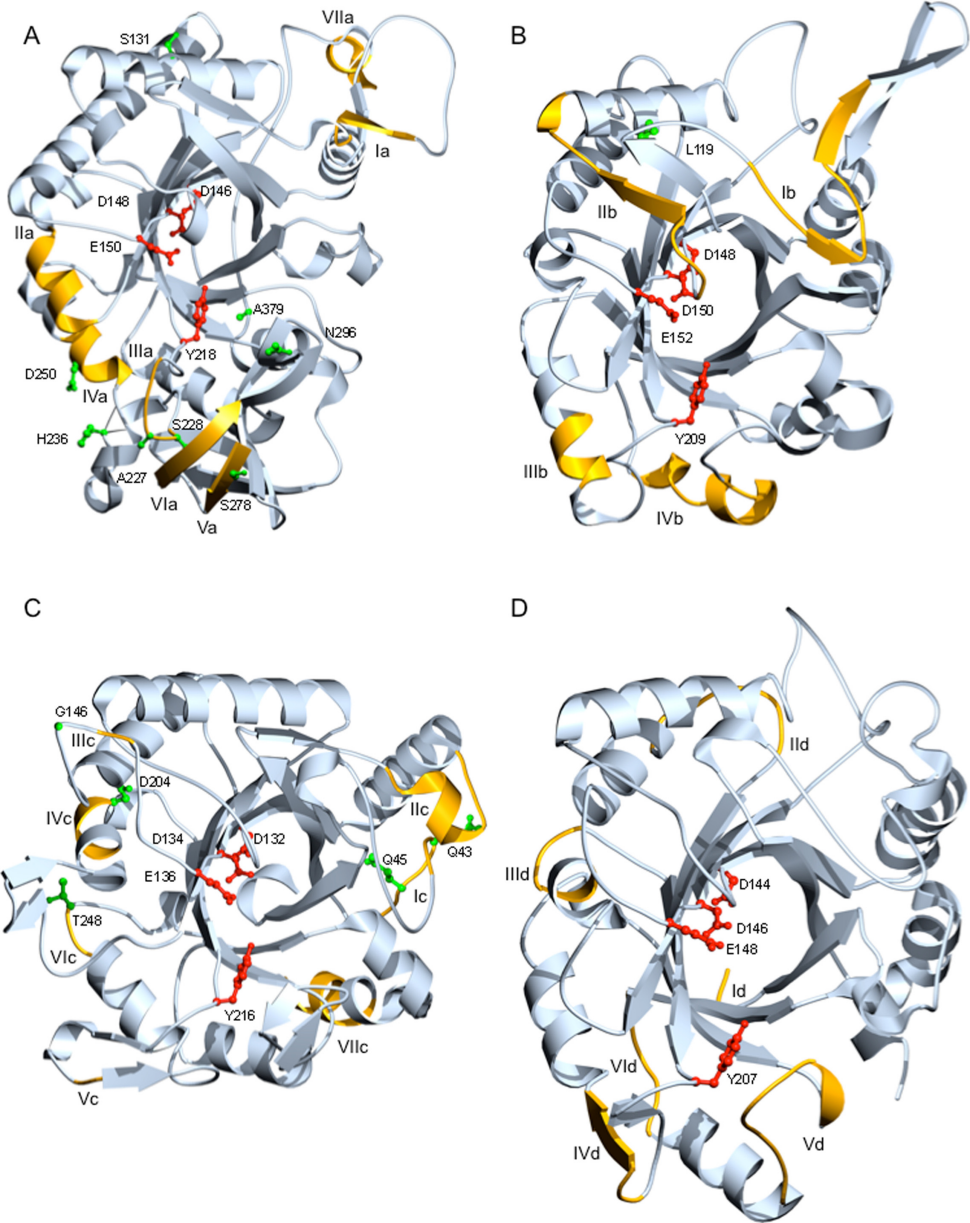
Homology models of *H. jecorina* chitinases. Homology models of the catalytic modules of *H. jecorina* chitinases (**A**) chi18-5, (**B**) chi18-13, (**C**) chi18-15 and (**D**) chi18-17 were generated using SOD and adjusted in O, based on hidden Markov models and Clustal W amino acid sequence alignments. Conserved catalytically important residues are indicated in red, amino acids under strong positive selection (Bayes factor ≥50) are indicated in green, variable regions from reverse conservation analysis (I scores ≥0.5) are indicated in orange and marked in Roman numerals from N- to C-termini. Residue numbering refers to catalytic module sequences used for modelling; 15–424 (chi18-5), 30–320 (chi18-13), 25–322 (chi18-15), 28–311 (chi18-17).

Amplification products and full-length sequences for *chi18-5* orthologs were successfully obtained from *H. schweinitzii*, *T. ghanense* and *T. longibrachiatum*. Additional sequences from *H. jecorina*, *H. atroviridis* and *H. virens* were retrieved from genome sequences and used for RCA analysis. A unique insert of 18 bp in *H. virens chi18-5* was excluded from the analysis. A phylogenetic analysis confirmed the orthologous status of the sequenced genes (Supplemental Figure S3). Amino acid diversity was distributed amongst eight regions with W mean scores above the 0.5 standard deviation threshold from the RCA analysis (Fig. 1). One of these regions was associated with the signal peptide cleavage site, while the other seven regions (Ia, IIa, IIIa, IVa, Va, VIa and VIIa) were visualized (Fig. 2A) using a homology model of *H. jecorina* chi18-5. Several of the twenty predicted residues (Supplemental Table S2) important for catalysis and substrate binding (cd06548 in Conserved Domain Database (CDD)^56^) were located in conserved regions with low W scores (Fig. 1). Three residues were located in regions with high W mean scores, one each in IIa, IIIa and IVa (Fig. 2A). These three regions, IIa, IIIa and IVa, were all surface-exposed and located on the product side of the enzyme. Region Ia was located in a loop that forms the entrance to the catalytic cleft, while region VIIa is on the surface of the enzyme but far from the catalytic site (Fig. 2A).

### Reverse conservation analysis of chi18-13 amino acid variability

**Figure 3.**
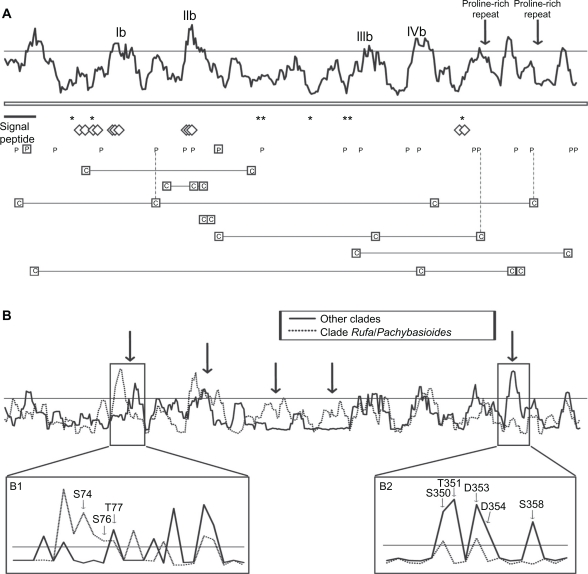
Reverse conservation analysis of chi18-13 orthologs and paralogs. **A**) Amino acid diversity was estimated using Rate4Site, based on a Clustal X alignment of chi18-13 *Trichoderma* orthologs and paralogs, and plotted as W mean scores. The y-axis represents arbitrary units (not shown) while a horizontal line indicates a 0.5 standard deviation cutoff. The x-axis represents residue position, asterisks (*) indicate positions of catalytic residues, diamonds (⋄) indicate substrate-interacting residues, boxed P indicate residues under strong (Bayes factor ≥50) positive selection, P indicate residues under weak (Bayes factor 10–49) positive selection, boxed C interconnected by horizontal lines indicate co-evolving residue groups and vertical dashed lines indicate identical residues. The position of the signal peptide, two proline-rich repeat units and regions with high amino acid diversity successfully visualised by homology modelling are indicated (Ib–IVb). **B**) Comparison of separate reverse conservation analyses on chi18-13 orthologs from *H. minutispora*, *H. parapilulifera*, *H. pilulifera* and *H. atroviridis* (dotted line) and *T. ghanense*, *H. jecorina*, *T. brevicompactum*, *H. citrina*, *H. schweinitzii*, *H. virens*, *T. tomentosum*, *H. lixii* and strain CBS816.68 (solid line). Arrows indicate regions with different W mean score distribution, magnifications illustrate residue S score distribution of the selected region.

Amplification products and full-length sequences for the *H. schweinitzii* and *H. virens chi18-13* orthologs were obtained, as were partial sequences presumably lacking the eight C-terminal residues from an additional nine species (Fig. S3). The *H. virens chi18-13* gene was sequenced in the current work because the ortholog from the genome sequence (protein ID 25421) lacked 70 C-terminal residues compared to translated chi18-13 orthologs from other *Trichoderma* species. Additional sequences from *H. jecorina* and *H. atroviridis* were retrieved from the genome sequences, together with two paralogous sequences, protein ID 79492 from *H. atroviridis* (originally cloned as Ech30)^10^,^14^ and 58102 from *H. virens*. Two short proline-rich repeat regions in the C-terminal part (reference pos. 320–337 and 373–380 in *H. atroviridis* 45585) were removed from all species before the analysis, due to the highly variable number of repeats between species. The two paralogous sequences, 79492 and 58102, were 70 C-terminal residues shorter than the orthologs. Phylogenetic analysis confirmed the orthologous and paralogous status of the selected sequences (Fig. S3). Amino acid diversity was distributed amongst eight regions with W mean scores above the 0.5 standard deviation threshold from the RCA analysis (Fig. 3A). Four regions (Ib, IIb, IIIb and IVb) were visualized by the homology model of *H. jecorina* chi18-13 (Fig. 2B). One of the non-mapped high W score regions was associated with the signal peptide cleavage site (Fig. 3A). The eight predicted residues important for catalysis and substrate-binding by homology modelling (Table S2) were located in conserved regions with low W scores (Fig. 3A). Predicted substrate-binding residues (cd02877 in CDD) were associated with regions Ib and IIb of low amino acid conservation levels (Fig. 3A). More specifically, substrate-binding residues S_74_, S_76_ and T_77_ (reference *H. atroviridis*) were located in region Ib, which forms the entrance to the catalytic cleft (Fig. 2B). Substrate-binding residues G_119_, A_120_ and V_121_ (reference *H. atroviridis*) were located in region IIb, which forms a loop that protrudes into the catalytic centre of chi18-13 (Fig. 2B). Regions IIIb and IVb were located on the surface but were not a part of the catalytic cleft (Fig. 2B).

Phylogenetic analysis of chi18-13 (Fig. S3) revealed two separate groups of orthologs; one consisting of species from the taxonomic clades of *Rufa* and *Pashybasioides*, 57 the other consisting of species from several other *Trichoderma* clades. Although these groups were not always recovered in alternative phylogenetic analyses using other parameters such as the JTT substitution model in combination with complete deletion of missing data (data not shown), RCA analyses performed on these two groups separately revealed five regions with amino acid conservation patterns indicative of differential adaptations between the two groups (Fig. 3B). A detailed analysis of the S scores for the individual amino acid positions revealed high S scores for substrate-interacting residues in region Ib in the *Rufa* and *Pashybasioides* clade (Fig. 3B). Low S scores were found for a region of unknown function situated between two proline-rich-repeats in the C-terminal part of chi18-13 in the *Rufa* and *Pashybasioides* clade, compared with the other species (Fig. 3B).

### Reverse conservation analysis of chi18-15 amino acid variability

**Figure 4.**
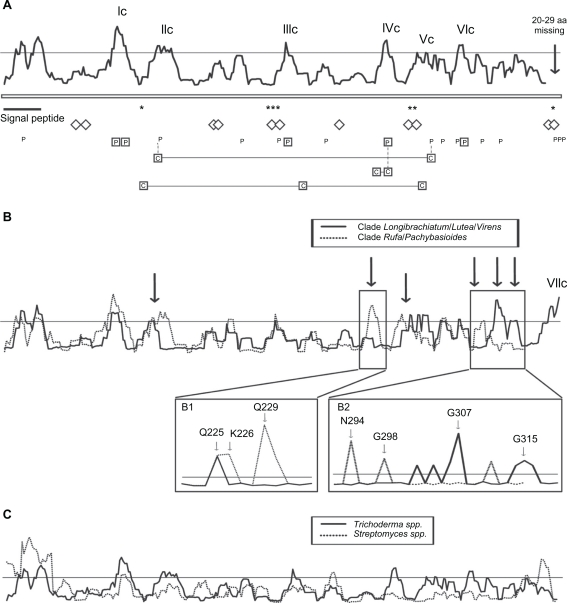
Reverse conservation analysis of chi18-15 orthologs. **A**) Amino acid diversity was estimated using Rate4Site, based on a Clustal X alignment of chi18-15 *Trichoderma* orthologs, and plotted as W mean scores. The y-axis represents arbitrary units (not shown) while a horizontal line indicates a 0.5 standard deviation cutoff. The x-axis represents residue position, asterisks (*) indicate positions of catalytic residues, diamonds (⋄) indicate substrate-interacting residues, boxed P indicate residues under strong (Bayes factor ≥50) positive selection, P indicate residues under weak (Bayes factor 10–49) positive selection, boxed C interconnected by horizontal lines indicate co-evolving residue groups and vertical dashed lines indicate identical residues. The positions of the signal peptide, a C-terminal region not included in the overall analysis and regions with high amino acid diversity successfully visualised by homology modelling are indicated (Ic–VIc). **B**) Comparison of separate reverse conservation analyses on chi18-15 orthologs from *H. minutispora*, *H. parapilulifera*, *H. pilulifera*, *H. atroviridis*, *H. rufa* and *T. croceum* (dotted line) and *T. ghanense*, *H. jecorina*, *T. brevicompactum*, *H. schweinitzii*, *H. virens* and *T. longibrachiatum* (solid line). Arrows indicate regions with different W mean score distribution, magnifications illustrate residue S score distribution of the selected region. **C**) Comparison of separate reverse conservation analyses on chi18-15 orthologs from *Trichoderma* species and ChiJ orthologs from *Streptomyces* species.

Amplification products and partial sequences for *chi18-15* orthologs, presumably lacking 20–29 amino acid residues in the C-terminal part, were obtained from nine different *Trichoderma* species (Fig. S3). Additional full-length sequences from *H. jecorina*, *H. atroviridis* and *H. virens* were retrieved from the genome sequences. A phylogenetic analysis confirmed the orthologous status of the sequenced genes (Fig. S3). Amino acid diversity was distributed amongst eight regions with W mean scores above the defined threshold (Fig. 4A). Additional analyses of sequences from taxonomic subgroups (see below) identified an additional region of high amino acid diversity in the C-terminal end of chi18-15 (Fig. 4B). Two of these regions were associated with the secretion signal peptide while the other seven regions (Ic, IIc, IIIc, IVc, Vc, VIc and VIIc) were located in the catalytic module shown in Figure 2C prepared using the homology model of *H. jecorina* chi18-15. Although several of these regions were surface-exposed in the homology model, none of the parts contribute to the catalytic cleft. The seven residues predicted as important for catalysis in chi18-15 (cd02871 in CDD) were all located in conserved regions with low W scores, as were all predicted substrate-interacting residues (Fig. 4A).

Phylogenetic analysis of chi18-15 (Fig. S3) revealed two separate groups of orthologs; one consisting of species from the taxonomic clades of *Rufa* and *Pashybasioides*, the other consisting of species from clades *Longibrachiatum*, *Lutea* and *Virens*.^57^ Again, some combinations of substitution models and handling of missing data in the phylogenetic analyses resulted in a more collapsed tree topology (data not shown), but the initial grouping was useful for more detailed analyses of amino acid variability. RCA analyses performed on these two groups separately revealed six regions with amino acid diversity patterns indicative of differential adaptations between the two groups (Fig. 4B). A detailed analysis of the S scores for the individual amino acid position revealed high conservation at position Q_229_ (ref. *H. atroviridis*) in the *Longibrachiatum*, *Lutea* and *Virens* clades, where all species contained an aspartic acid residue while the position was occupied by either aspartic acid, glutamine or glutamic acid in the *Rufa* and *Pashybasioides* clade (Fig. 4B). Three other positions that displayed amino acid variability in either group were pos. N_294_, G_298_ and G_307_ (ref. *H. atroviridis*) in the C-terminal part of chi18-15 (Fig. 4B).

*H. jecorina* chi18-15 was previously shown to be of actinobacterial origin and horizontally transferred to *Trichoderma*,^12^ most closely related to ChiJ from *S. coelicolor*. Therefore, a separate RCA analysis was performed on an alignment of six orthologs of ChiJ from *S. coelicolor*, *S. avermitilis*, *S. clavuligerus*, *S. ghanaensis*, *S. griseus* and *S. sp.* Mg1 (Supplemental Table S3). The result showed that the amino acid diversity in *Streptomyces* ChiJ was distributed at different position than among the *Trichoderma* chi18-15 orthologs (Fig. 4C).

### Reverse conservation analysis of chi18-17 amino acid variability

**Figure 5.**
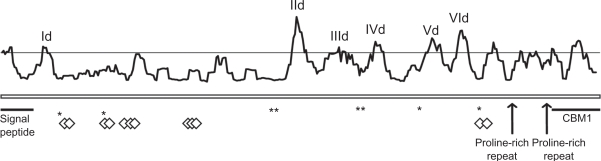
Reverse conservation analysis of chi18-17 orthologs. Amino acid diversity was estimated using Rate4Site, based on a Clustal X alignment of chi18-17 *Trichoderma* orthologs, and plotted as W mean scores. The y-axis represents arbitrary units (not shown) while a horizontal line indicates a 0.5 standard deviation cutoff. The x-axis represents residue position, asterisks (*) indicate positions of catalytic residues, diamonds (⋄) indicate substrate-interacting residues. The positions of the signal peptide, two proline-rich repeat units, a CBM1 substrate-binding region and regions with high amino acid diversity successfully visualised by homology modelling are indicated (Id–VId).

Amplification products and full-length sequences for *chi18-17* orthologs were successfully obtained from *H. schweinitzii*, *T. ghanense*, *T. tomentosum* and *H. lixii*, and additional sequences from *H. jecorina*, *H. atroviridis* and *H. virens* were retrieved from the genome sequences. Two introns were present in *H. jecorina*, *H. schweinitzii* and *T. ghanense*, compared with only one intron in *H. atroviridis*. No intron was present in *H. virens*, *T. tomentosum* or *H. lixii*, although in one isolate of *H. lixii* (CBS275.78) a unique insert was present that was interpreted as an intron and thus excluded from the analysis. A phylogenetic analysis confirmed the orthologous status of the sequenced genes (Fig. S3). Two short proline-rich repeat regions in the C-terminal part (pos. 324–331 and 350–355 in *H. atroviridis*) were excluded from the analysis, because the number of repeats was highly variable between species. Amino acid diversity was distributed amongst 13 regions with W mean scores above the defined threshold (Fig. 5). One of these regions was associated with the secretion signal peptide while two were situated in a C-terminal family 1 Carbohydrate-Binding Module (CBM1, cellulose and chitin binding), more specifically in the β1 and β2 antiparallel β-sheets (pos. 360–364 and 379–383 in *H. atroviridis*) (Fig. 5). Six regions were visualized using the homology model of *H. jecorina* chi18-17, (Id, IId, IIId, IVd, Vd and VId) (Fig. 2D). Region Vd formed a part of the catalytic cleft while the other five were predicted to be surface-exposed but not directly associated with the catalytic cleft (Fig. 2D). The eight predicted residues important for catalysis and substrate-binding (cd02877 in CDD) were all located in conserved regions with low W scores (Fig. 5).

### Analysis of codon-usage

**Figure 6.**
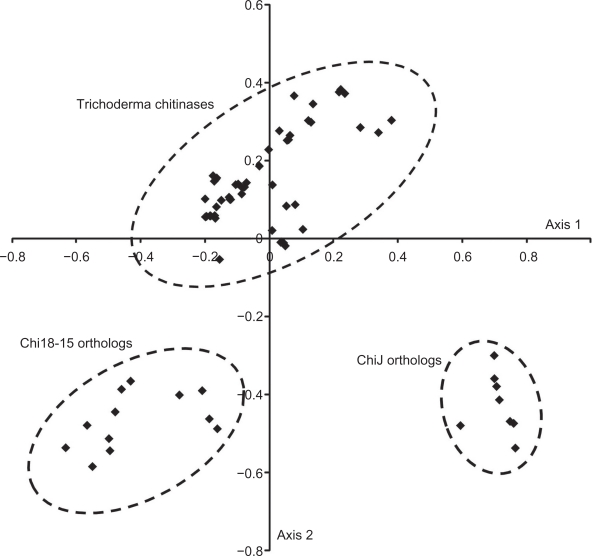
Codon-usage of *Trichoderma* chitinases and *Streptomyces ChiJ* orthologs. Correspondence analysis of codon-usage was performed on *Trichoderma chi18-5*, *chi18-13*, *chi18-15*, *chi18-17* orthologs and *Streptomyces ChiJ* orthologs, using the program CodonW accessed through the Mobyle web interphase. The resulting coa-axis 1 and 2 (in arbitrary units) for codon-usage for each gene was plotted in Excel. Dashed circles indicate groups of *chi18-15* orthologs, other *Trichoderma* chitinases and *ChiJ* orthologs.

Adaptation of codon-usage in *Trichoderma* chitinases and *Streptomyces ChiJ* orthologs was investigated by correspondence analysis of codon-usage using CodonW.^37^ Plotting coa-axis 1 and 2 for codon-usage for each gene identified three different clusters representing *chi18-15* orthologs, other *Trichoderma* chitinases and *Streptomyces ChiJ* orthologs (Fig. 6).

### Codon-based likelihood analyses

The mean pair wise nucleotide identity percentages among the four *Trichoderma* chitinases was; 84.6 ± 4.4 (standard deviation) for *chi18-5*, 88.6 ± 4.0 for *chi18-13*, 80.3 ± 6.1 for *chi18-15* and 83.1 ± 6.3 for *chi18-17*. Only *H. jecorina*, *H. atroviridis*, *H. virens*, *T. ghanense* and *H. schweinitzii* were included in this comparison, as data for only these species were available for all four genes.

In order to study the mechanisms behind the observed patterns of amino acid variability, we used REL analysis^48^ to test for the presence of codons under different evolutionary constraints and to identify them. As recommended when using REL, ten species representatives were considered to be the minimum number of sequences for this analysis to provide reliable results; only *chi18-13* and *chi18-15* fulfilled this requirement. Two short proline-rich repeat regions in the C-terminal part of chi18-13 were removed before REL analysis as the number of repeats was highly variable between species. REL fits both dN and dS substitution rates into three discrete distributions, yielding a total number of nine different rate classes of dN/dS. For *chi18-13*, one rate class was estimated to have dN/dS values above 1 (dN (0.49)/dS (0.21) = 2.33). A similar result was obtained for *chi18-15* where one rate class was estimated to have dN/dS values above 1 (dN (0.80)/dS (0.67) = 1.20). Using a cutoff of a Bayes factor ≥50, two sites in *chi18-13* were identified as displaying signatures of positive selection (Table 2), 64 displayed signatures of purifying selection, and 324 evolved neutrally. One of the positively selected sites was located in the signal peptide (pos. T_16_, ref. *H. atroviridis*). The other site was closely located to region IIb (pos. V_137_, ref. *H. atroviridis*), which was modelled to protrude into the catalytic centre of chi18-13 (Fig. 2B). Furthermore, this second positively selected site coincided with one region with very different patterns of amino acid variability (W means) between chi18-13 orthologs from the *Rufa*/*Pashybasioides* and other clades (Fig. 3A and 3B). Between a Bayes factor of 10 and 49, an additional 17 sites displayed weak signatures of positive selection (Fig. 3A). For *chi18-15*, using a cutoff of a Bayes factor ≥50, five sites were identified as displaying signatures of positive selection (Table 2), 204 displayed signatures of purifying selection and 135 evolved neutrally. Between a Bayes factor of 10 and 49, an additional 13 sites displayed weak signatures of positive selection (Fig. 4A). All five positively selected sites were located in regions with high amino acid diversity, identified by RCA, pos. I_68_ and K_70_ (ref. *H. atroviridis*) in region Ic, pos. N_171_ in IIIc, pos. Q_229_ in IVc and pos. T_273_ in VIc (Fig. 4A).

For comparative purposes, REL analysis was performed on partial sequences of two *Trichoderma* genes, the functions of which were assumed to be independent from mycoparasitic interactions, actin (*act*) and translation elongation factor 1 alpha (*tef* ). These sequences were retrieved from GenBank (Table S3), and included 627 bp for *act* (pos. 2-628 in *H. virens*, acc. no. FJ442590) and 228 bp for *tef* (pos. 132–158, 258–320, 613–750 in *H. virens*, acc. no. EU280065). No sites displayed signs of positive selection (Bayes factor ≥50) in either gene.

### Analysis of co-evolving codons

Amino acid residues can interact structurally with each other to form and stabilize protein structures, or interact functionally through participation in the same protein function, such as substrate binding and processing. Therefore, co-evolution between sites in *chi18-13* and *chi18-15* were studied using evolutionary-network models implemented in Spidermonkey/BGM.^55^ In *chi18-13* thirteen interacting pairs of codons were identified (Table 3), forming seven groups of co-evolutionary sites (Fig. 3A). Three interacting groups included sites located in or close to region IIb, while three other groups included sites that were associated with regions IIIb or IVb. In three cases the interacting residues also showed weak signatures of positive selection (Fig. 3A). In *chi18-15*, four interacting pairs of codons were identified (Table 3), forming three groups of co-evolving sites (Fig. 4A). In one case, the interacting residue also displayed strong signatures of positive selection, and in two cases the interacting residues showed weak signatures of positive selection (Fig. 4A).

## Discussion

The complete genome sequence of three different *Trichoderma* species, *H. jecorina*, *H. atroviridis* and *H. virens* revealed the complexity of the chitinase enzyme system in these species illustrated by a total number of 20, 29 and 36 different chitinase genes respectively. The size of the chitinase gene family in the two mycoparasitic species *H. atroviridis* and *H. virens*, indicates that hydrolytic breakdown of the antagonists cell walls is important during the mycoparasitic interaction. However, chitinases are also involved in other functions such as morphological development, sporulation and autolysis.^9^ Studying phenotypic effects in gene knock-out approaches is less likely to reflect the true biological function of a chitinase because of compensatory effects from paralogs, illustrating the need for complementary approaches.

Certain plant defence chitinases from the genus *Arabis* and the family *Poaceae* evolved rapidly in response to a co-evolutionary arms race between plant host and fungal pathogen, resulting in a continuous selection for adaptive modifications.^19^,^20^ Conceptually, the same situation may apply to microbe-microbe interactions; hence the combination of specific expression patterns during mycoparasitism and adaptive evolutionary changes may provide important information when assigning biological functions to *Trichoderma* chitinases.

### Chitinase gene family expansion

In the mycoparasitic species *H. atroviridis* and *H. virens* subgroups B1/B2 and C1/C2 have expanded significantly in paralog numbers. Stress-related genes often exhibit many expansions and contractions during fungal evolution; 58 hence the observed expansion suggest a role of at least some *Trichoderma* B1/B2 and C1/C2 subgroup members in aggressive fungal-fungal interactions. Gene duplications may relieve selective constraints on one gene copy which can evolve modified substrate specificities or enzyme properties more adapted towards specific cell wall composition in antagonistic species. Expansions of subgroups B1/B2 and C1/C2 are also observed in other soil-borne ascomycetes such as *Gibberella zeae*, *Uncinocarpus reeseii* and *Emericella nidulans*, while gene copy number in these subgroups is reduced in the human pathogen *Coccidioides immitis*.^7^

### Evolution of chitinase chi18-13

*Chi18-13* is a member of the B1/B2 subgroup and displays the highest mean nucleotide conservation level among the studied chitinases. However, several codons are predicted to evolve under positive selection or form co-evolutionary site groups. Amino acid diversity is distributed amongst four successfully modelled regions, where Ib and IIb form parts of the catalytic cleft. This suggests that adaptations for substrate-specificity may be an important aspect for chi18-13 evolution. Three co-evolving groups of amino acid sites are associated with region IIb, supporting three substrate-interacting residues. It is possible that the observed site co-evolution is the result of modifications in the position of the substrate-interacting residues at optimal distances from the substrate in different *Trichoderma* species. In addition, three other interacting groups are associated with regions IIIb and IVb, and these co-evolving groups also include sites that are located in other parts of chi18-13, especially in the C-terminal part close to two proline-rich repeat units of variable lengths between species. These repeats can possibly function as linkers to provide flexibility in the secondary structure of chi18-13. This suggests that chi18-13 processivity, in addition to substrate-specificity, has been under selection for modification during chi18-13 evolution. Processivity may be influenced by distant parts of chi18-13 and not merely by the loops that constitutes the catalytic cleft.

Expression data for the *H. atroviridis chi18-13* paralog *Ech30* (prot. ID 79492) show that the gene is induced by fungal cell wall material and during plate confrontation assays.^10^,^14^ Enzyme activity of *H. atroviridis* Ech30 shows that it is an endochitinase with preferential activity towards longer substrates, such as chitin fibre.^14^,^59^ The low activity against short substrates suggests a shallow catalytic cleft for both Ech30 and chi18-13 which is in agreement with our modelling data. In summary, evolutionary data identify chi18-13 as a candidate enzyme for mycoparasitic attack. Chi18-13 is a member of a paralog cluster in mycoparasitic *H. atroviridis* and *H. virens* and displays signs of accelerated rates of evolution. Amino acid variability and co-evolution among sites of chi18-13 are associated with regions that are not only predicted to influence substrate-specificity and processivity, but which also display differences in variability patterns between *Trichoderma* clades.

### Evolution of chitinase chi18-15

The optimal codon-usage of *chi18-15* is different from codon-usage in other *Trichoderma* chitinases and from the *Streptomyces ChiJ* gene. We previously demonstrated through phylogenetic analysis of the chitinolytic domain that *H. jecorina chi18-15* was introduced into the ancestor of *Trichoderma* through horizontal transfer from an actinobacterial origin.^12^

Two sites that evolved under positive selection are located in region Ic, which is located on the substrate entrance side of the protein, but not part of the catalytic cleft. In addition, two groups of co-evolutionary sites are located in both regions IIc and Vc which suggests concurrent structural adaptations of both substrate- and product sides of chi18-15. However, since neither region of high amino acid diversity (Ic to VIIc) is modelled to directly form part of the catalytic cleft, one interpretation is that they are the result of random mutations accumulating at regions with low selective constrains. The alternative explanation is that these regions are indeed important for functional properties of chi18-15 and this explanation is supported for regions IVc and VIc. Both regions contain sites under accelerated evolutionary rates and display discreet differences between different *Trichoderma* clades or between *Streptomyces* ChiJ orthologs, which is not compatible with a completely stochastic process. In addition, region IVc contains two sites that co-evolved, again suggesting functional relevance for this region whether it be the maintenance of secondary structures, interaction with enhancer- or inhibitor proteins, or additional unknown functions.

Another region that displayed differences between *Trichoderma* clades contained site G_298_ (ref. *H. atroviridis*) which is shown to determine activity inhibition by the chitinase inhibitor kinetin.^45^ In *S. cerevisiae* CTS1, changing the alanine in this site to a bulkier serine residue eliminates inhibition by kinetin, whereas inhibition by allosamidin, acetazolamide and 8-chlorotheophylline remain unchanged.^45^ It is possible that the observed differences between *Trichoderma* clades in this region represents an adaptation towards differences in antagonist inhibitor counter-measures. In comparison, the homologous position in chi18-5, chi18-13 and chi18-17 all contain a bulky methionine residue that may abolish inhibition.^45^ This difference may reflect a recent introduction of chi18-15 into *Trichoderma* and ongoing adaptations towards fungal preferences.

*Chi18-15* gene expression is induced by a variety of stimuli, including chitin and its monomers, nitrogen starvation, temperature and osmotic stress, and by interaction with *Rhizoctonia solani*.^16^ Chi18-15 has been reported to possess endochitinase activity with acidic optima and preferential activity towards high molecular weight substrates.^16^,^60^ As with chi18-13, evolutionary data identify chi18-15 as a candidate enzyme for mycoparasitic attack. The gene has its ancestral origin as an actinobacterial chitinase, presumably an aggressive component in bacterial-fungal interactions. After being horizontally transferred into *Trichoderma*, it has been subjected to strong selective pressures to modify its functional properties according to the specific ecological contexts of different *Trichoderma* species.

### Evolution of chitinase chi18-5

In chi18-5, the observed amino acid diversity is preferentially situated in regions on the product side of the catalytic cleft that probably interact with substrate cleavage products as they leave the catalytic site. Therefore, these regions may be involved in determining the processivity of the enzyme. Regions IIa, IIIa and IVa are associated with residues that interact with the substrate at subsite +2, which has been confirmed by earlier studies from *T. harzianum* chi18-5.^61^ The model also confirms the deep catalytic cleft of chi18-5 which provides tight binding of the substrate.^61^

The *Chi18-5* orthologs are induced by chitin degradation products and during mycoparasitic interactions,^10^,^15^,^18^,^62^–^65^ but gene knock-out experiments are inconclusive. Two reports show no reduction in the ability of *H. atroviridis* to overgrow other fungi in plate confrontation assays,^66^,^67^ while one study showed a reduction of *H. virens* biocontrol ability.^68^ Thus the function of chi18-5 orthologs is suggested to be degradation of exogenous chitin for nutritional needs rather than a direct involvement in mycoparasitism.^9^ This function does not contradict our evolutionary data; a conserved enzyme with a deep catalytic cleft that can bind chitin tightly. Slight modifications between different *Trichoderma* species may be associated with processivity but not with substrate specificity.

### Evolution of chitinase chi18-17

In chi18-17, the two regions of high amino acid diversity that are located in the CBM1 substrate-binding module suggest that there are discrete modifications of the binding properties of the chi18-17 CBM1 domain in different *Trichoderma* species. A similar example is found in a plant defence chitinase where positively selected amino acid positions are located in a substrate-binding module.^20^ Only one variable region (Vd) is part of the catalytic cleft, which is wider and shallower than in chi18-5, while all other regions identified by RCA are predicted to be surface-exposed but not directly associated with the catalytic cleft. Interpretation of these regions is highly speculative; they may interact with other proteins in the environment, or alternatively they may represent regions of low selective constrains where a limited amount of amino acid variability is tolerated. *H. virens chi18-17* is reported to be induced by fungal cell wall material but in depth studies are missing.^15^ The overall picture from evolutionary data depict a conserved protein with a shallow catalytic cleft, indicating endo-activity, without any obvious changes in known functional regions which suggests conserved enzymatic properties between *Trichoderma* orthologs.

### Concluding remarks

Certain plant defence chitinases from the genus *Arabis* and the family *Poaceae* have evolved rapidly in response to a co-evolutionary arms race between plant host and fungal pathogen, resulting in a continuous selection for adaptive modifications.^19^,^20^ Under these conditions, only a limited number of sites evolved under positive selection as severe structural constraints are present in chitinases to preserve catalytic function. This is observed also for fungal chitinases in the current study. In chi18-13 and chi18-15 only a few sites are identified to evolve under positive selection or to co-evolve with other sites. In addition, we detected a number of regions that display high amino acid diversity without any signs of accelerated evolution. Although one explanation may be low selective constraints in these regions, the localization to substrate- or product side of the catalytic cleft and differences in variability/conservation patterns between different *Trichoderma* clades, suggests that amino acid variation between species in at least some of these regions represents adaptive modifications.

The observed evolutionary differences between chi18-5, chi18-13, chi18-15 and chi18-17, together with data on different domain-structures, different expression patterns and different enzymatic properties support the idea of functional differentiation between fungal chitinases. Therefore, correct functional assignment of individual genes and proteins are vital for a proper mechanistic understanding of biocontrol. Using data on molecular evolution in a fungal-fungal interaction framework is one possible approach that can aid our understanding of mycoparasitism and structure/function relationships in enzymes.

## Supplemental Materials

Figure S1. Distribution of chitinase gain and loss among fungal lineages. Phylogenetic relationships among the fungal species used in the current study are shown, including divergence dates in millions of years. Circled numbers represent total number of chitinase genes in extant species and estimates of total number of chitinase genes for ancestral species. Boxed taxon names indicates a significant (P-values ≤ 0.05 or Likelihood ratios ≥ 50) expansion (+), or a significant contraction (−) of the chitinase gene family size.

Figure S2 Group A. Phylogeny of group A Trichoderma chitinases. Analysis was performed using neighbour-joining implemented in MEGA version 4 with the Poisson correction of substitution rates and complete deletion of missing data, based on a Clustal W alignment of chitinase catalytic domain amino acid sequences. Branch support values (bootstrap proportions ≥ 60) are associated with nodes. The bar marker indicates numbers of amino acid substitutions. Protein identifiers include protein name (if available) or protein ID nos. from the respective genome projects. Subgroup names are indicated.

Figure S2 Group B. Phylogeny of group B Trichoderma chitinases. Analysis was performed using neighbour-joining implemented in MEGA version 4 with the Poisson correction of substitution rates and complete deletion of missing data, based on a Clustal W alignment of chitinase catalytic domain amino acid sequences. Branch support values (bootstrap proportions ≥ 60) are associated with nodes. The bar marker indicates numbers of amino acid substitutions. Protein identifiers include protein name (if available) or protein ID nos. from the respective genome projects. Group names are indicated.

Figure S2 Group C. Phylogeny of group C Trichoderma chitinases. Analysis was performed using neighbour-joining implemented in MEGA version 4 with the Poisson correction of substitution rates and complete deletion of missing data, based on a Clustal W alignment of chitinase catalytic domain amino acid sequences. Branch support values (bootstrap proportions ≥ 60) are associated with nodes. The bar marker indicates numbers of amino acid substitutions. Protein identifiers include protein name (if available) or protein ID nos. from the respective genome projects. Group names are indicated.

Figure S3. Trichoderma chitinase gene phylogenies. Analyses were performed on chitinases (**A**) chi18-5, (**B**) chi18-13, (**C**) chi18-15 and (**D**) chi18-17 using neighbour-joining implemented in MEGA version 4 with the Poisson correction of substitution rates and complete deletion of missing data, based on a Clustal W alignment of chitinase amino acid sequences. Branch support values (bootstrap proportions ≥ 60) are associated with nodes. The bar marker indicates numbers of amino acid substitutions. Protein identifiers include protein name (if available) or protein ID nos. from the respective genome projects.

**Table S1.** Oligonucleotide primers used in the current study.

**Gene**	**Oligonucleotide**	**Sequence (5′→3′)**
*Chi18-5*	Chi18-5.F1	AAGCACTATGCsGATGATT
	Chi18-5.F2	GGTTACmTGTyrkTGCCAAG
	Chi18-5.F3	CAACGTTGCTAsACTTGG
	Chi18-5.F4	TGAAGGAyTGGGGyTTyG
	Chi18-5.F5	GTTCCCGCAArCAAGATT
	Chi18-5.F8	GGTrTCTGGGAyTACAAGG
	Chi18-5.R1	CTGTATATACgTrTkTGCCTATG
	Chi18-5.R2	ArCAGAAGrATCATGTTGG
	Chi18-5.R3	CAyTsGCTCTCTTCTCAAC
	Chi18-5.R4	GwAAGkCTGGCCAATrCCAG
	Chi18-5.R6	GCCTATGTACAGTGGTATATGTG
*Chi18-13*	Chi18-13.F1	AGCGCTTCAAGTCCAACT
	Chi18-13.F2	GGAGCCACCTTCCGGATT
	Chi18-13.F4	ATGwTCTTCAGCAAAGCwCT
	Chi18-13.F6	TGGGTCCAGTTyTACAAyAA
	Chi18-13.R1	CGGGAACmCATGATGACAC
	Chi18-13.R2	CTGrGCyTCCCArAGCAT
	Chi18-13.R3	GTTGTAGAACTGrACCCAGATGT
	Chi18-13.R4	GAGCAGACrCCGTTCTTG
*Chi18-15*	Chi18-15.F2	CwTwmrGAmATCCTACGTTAC
	Chi18-15.F3	TGArwGAATACTACCTTCTCrA
	Chi18-15.F4	ACAAmACGGCTACAACGTGA
	Chi18-15.F5	GGCGTyCTyGCrCAGATG
	Chi18-15.F6	ATCGACATTGACATCGAGAC
	Chi18-15.F7	GTTCCCTAyAryATGCAA
	Chi18-15.F8	TACAGCAyTATGGGAArACG
	Chi18-15.R1	GCCGTTGTCGACGTATTTCT
	Chi18-15.R3	CryCsGCwyTCTTCyrGTATCA
	Chi18-15.R4	ACTTTmACAGTrGCrTCCAT
	Chi18-15.R5	CrTCCCAGTTGATwGACCA
	Chi18-15.R6	TGTAATGCTACCACCTGTGA
	Chi18-15.R7a	GTGAyrAmTTTATATACTG
	Chi18-15.R7b	CAGAGCAmyCATACATGTCG
	Chi18-15.R8	CCATAGTCAAGCCAAAGT
*Chi18-17*	Chi18-17.F1	ATTCATGTAACCATGTCAG
	Chi18-17.F2	GCsArTyCAGAGCTGTCT
	Chi18-17.F3	AGACvATCATGATGAGTCTG
	Chi18-17.F4	TCATGCTGTGGGATATGG
	Chi18-17.R1	AAGkGAATATwTACAAACAGAA
	Chi18-17.R2	CCGTTGTTGTAGAACTGAAC
	Chi18-17.R3	CCAACCATGAAGGArATGT
	Chi18-17.R4	AATCTTGGCCAGGTATCC

**Table S2.** Residues of *Trichoderma* models corresponding to the catalytic and substrate binding residues of 1FFR (*Serratia marcescens* Chitinase A) structure. Residue numbering refers to catalytic module sequences used for modelling; 15–424 (chi18-5), 30–320 (chi18-13), 25–322 (chi18-15), 28–311 (chi18-17).

**Site**	**1FFR**	**Chi18-5**	**Chi18-13**	**Chi18-15**	**Chi18-17**
Active site	D313	D148	D150	D146	D134
	E315	E150	E152	E148	E136
	F/Y390	Y218	Y209	Y207	Y216
	D311	D146	D148	D144	D132
−5	Y170	Y29	—	—	—
−4	R172	R31	—	—	—
−3	W167	W26	—	—	W23
	T276	T111	V103	—	T101
	E473	E295	—	—	—
−2	W275	W110	A102	S98	A100
	T276	T111	V103	S98	T101
	E473	E295	—	—	—
	W539	W357	W279	W281	W298
	E540	E358	E280	D282	S299
−1	Y163	Y22	Y16	Y13	Y19
	W275	W110	A102	S98	A100
	D313	D148	D150	D146	D134
	E315	E150	E152	E148	E136
	A362	A190	A183	A179	A175
	M388	M216	Q207	Q205	Q214
	F/Y390	Y218	Y209	Y207	Y216
	D391	D219	N210		
	Y444				
	R446	R274	?	—	—
	W539	W357	W279	W281	W298
+1	W275	W110	A102	S98	A100
	E315	E150	E152	E148	E136
	F316	Y151			
	M388	M216	Q207	Q205	Q214
	D391	D219	N210		
	R446	R274	?	—	—
+2	W275	W110	A102	S98	A100
	K369	N197	—	—	—
	D391	D219	N210		
	F396	W224	—	—	—
	Y418	F246	N213	G210	—

**Table S3.** Species and acc. nos. for gene sequences retrieved from GenBank, and used in the current work.

**act Species**	**GenBank acc.nos.**	**tef Species**	**GenBank acc.nos.**	**ChiJ Species**	**GenBank acc. nos.**
*Hypocrea flaviconidia*	DQ111960	*H. andinensis*	EU280042	*Streptomyces avermitilis*	NP_826813
*H. lutea*	FJ442602	*H. crassa*	EU280053	*S. clavuligerus*	ZP_05007692
*H. melanomagna*	FJ442601	*H. koningii*	EU280017	*S. ghanaensis*	ZP_04683727
*H. minutispora*	DQ111977	*H. lixii*	EU279994	*S. griseus*	YP_001828239
*H. pachybasioides*	DQ111976	*H. novaezelandiae*	EU280039	*S. sp. Mg1*	ZP_04999219
*H. rufa*	DQ333563	*H. orientalis*	EU280038	*S. coelicolor*	NP_626743
*H. stilbohypoxyli*	DQ111967	*H. tawa*	EU279972		
*H. virens*	FJ442590	*H. virens*	EU280065		
*H. viridescens*	FJ442594	*H. lutea*	EU280058		
*Trichoderma asperellum*	EU856272	*T. asperellum*	EU279961		
*T. austrokoningii*	DQ379011	*T. atroviride*	EU280024		
*T. caribbaeum*	DQ328610	*T. brevicompactum*	EU280061		
*T. dingleyeae*	DQ367718	*T. citrinoviride*	EU280036		
*T. dorotheae*	DQ379009	*T. cuenisporum*	EU280052		
*T. erinaceum*	DQ323450	*T. gamsii*	EU280005		
*T. evansii*	EU856269	*T. ghanense*	EU280043		
*T. hamatum*	EU856267	*T. helicum*	EU280055		
*T. koningiopsis*	DQ379014	*T. koningiopsis*	EU280012		
*T. lieckfeldtiae*	EU856276	*T. longipile*	EU280051		
*T. ovalisporum*	DQ328611	*T. ovalisporum*	EU280004		
*T. ovalisporum*	DQ328608	*T. pleuroticola*	EU279973		
*T. pauculosporum*	DQ111957	*T. pleurotum*	EU279975		
*T. petersenii*	DQ379013	*T. rossicum*	EU280066		
*T. pubescens*	EU856249	*T. saturnisporum*	EU280044		
*T. rogersonii*	DQ367716	*T. sinensis*	EU280041		
*T. spirale*	FJ442819	*T. spirale*	EU280050		
*T. strigosum*	DQ111964	*T. tomentosum*	EU279971		
*T. taiwanense*	DQ323455				
*T. theobromicola*	EU856270				
*T. viride*	DQ111970				

## Figures and Tables

**Table 1.** Fungal strains used in the current study.

**Species**	**CBS number**	**Geographical origin**
*H. citrina*^a^	593.76	Netherlands
*H. lixii*^a^	102174	Spain
*H. lixii*^a^	275.78	Colombia
*H. minutispora*^a^	341.93	Canada
*H. parapilulifera*^a^	112264	Australia
*H. pilulifera*^a^	224.84	Germany
*H. rufa*^a^	349.92	USA
*H. schweinitzii*^a^	258.85	USA
*H. virens*^a^	249.59	USA
*T. brevicompactum*^a^	109720	USA
*T. croceum*^b^	337.93	Canada
*T. ghanense*^a^	259.85	Canada
*T. longibrachiatum*^a^	182.69	Netherlands
*T. tomentosum*^a^	349.93	Canada
*Unidentified Trichoderma*	816.68	USA

a Based on ITS sequencing and TrichoOKey.

b Based on morphological characters by Centraalbureau voor Schimmelcultures.

**Table 2.** Positively selected sites in chi18-13 and chi18-15.

**Protein**	**Amino acid position^a^**	**Posterior Probability^b^**	**Bayes factor^b^**
Chi18-13	T_16_	0.88	52
	V_137_	0.97	213
Chi18-15	I_68_	0.69	62
	K_70_	0.80	111
	N_171_	0.81	119
	Q_229_	0.65	54
	T_273_	0.71	69

a Site position in reference to *H. atroviridis*.

b Determined by Random Effects Likelihood method.

**Table 3.** Co-evolving sites in chi18-13 and chi18-15.

**Protein**	**Amino acid position^a^**	**Amino acid position^a^**	**Posterior probability^b^**
Chi18-13	S_51_	T_159_	0.84
	T_97_	A_9_	0.79
	T_97_	N_281_	0.71
	V_121_	Q_125_	0.63
	S_129_	G_130_	0.86
	S_132_	I_312_	0.64
	S_132_	T_241_	0.72
	G_230_	N_369_	0.90
	Y_270_	S_332_	0.89
	N_281_	K_347_	0.83
	S_332_	L_18_	0.60
	D_335_	L_18_	0.56
	V_121_	K_104_	0.58
Chi18-15	S_86_	A_178_	0.59
	V_90_	A_254_	0.65
	Q_229_	Q_225_	0.59
	S_249_	A_178_	0.56

a Site position in reference to *H. atroviridis*.

b For site 1 and site 2 to be conditionally dependent, determined by Spidermonkey/BGM.

## Abbreviations

CAFE: Computational analysis of gene family evolution
CBM: Carbohydrate-binding module
CDD: Conserved domain database
dN: Non-synonymous substitution rate
dS: Synonymous substitution rate
ITS: Internal transcribed spacer
MEGA: Molecular evolutionary genetics analysis
PDB: Protein data bank
RCA: Reverse conservation analysis
REL: Random effects maximum likelihood models
