# Supplementary figures and images for: Comparative Molecular Evolution of Trichoderma Chitinases in Response to Mycoparasitic Interactions
Source: Evol Bioinform Online. 2010 Mar 15;6:1–26. doi: 10.4137/ebo.s4198 (PMC2865166; doi:10.4137/ebo.s4198)

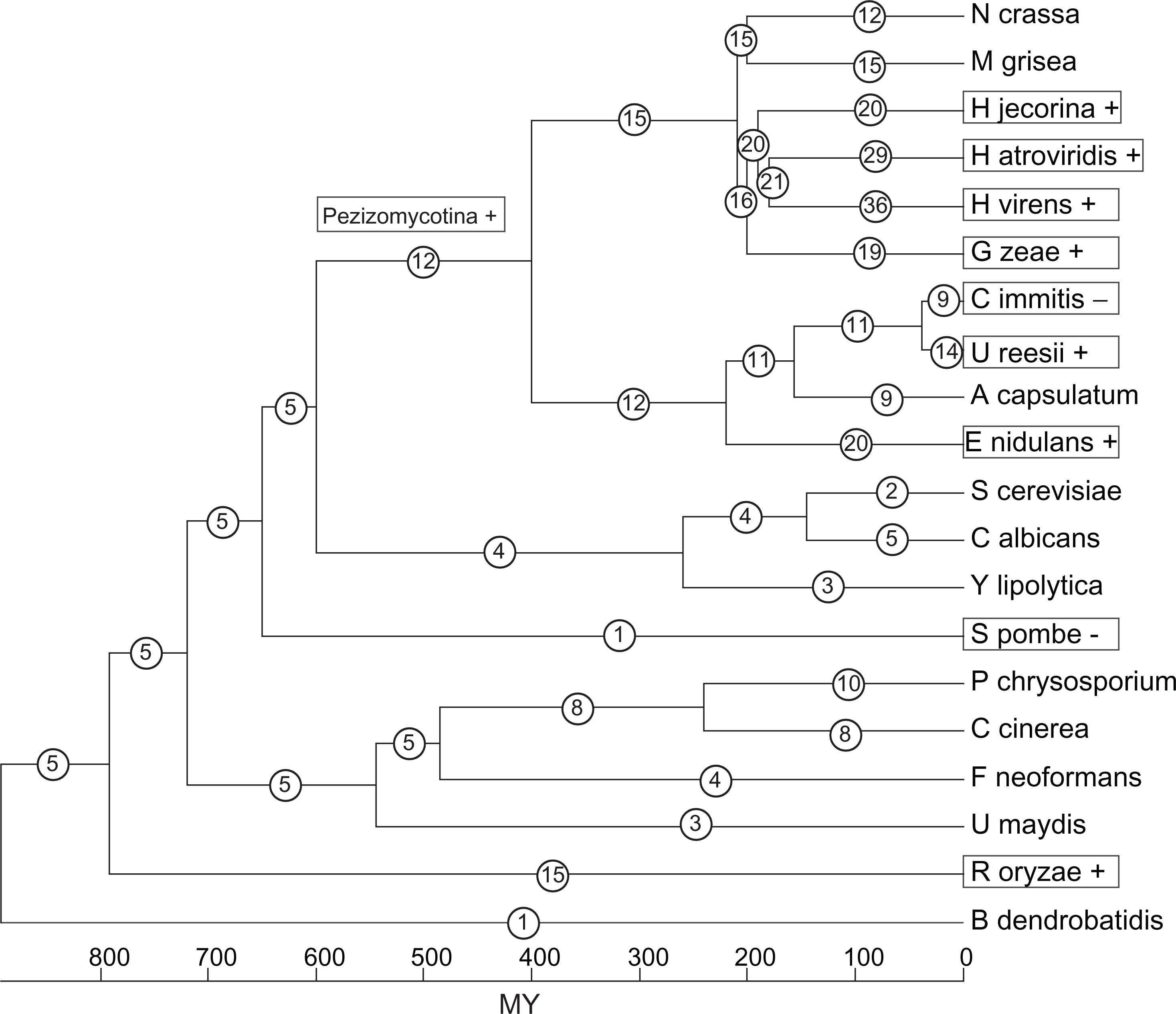

Supplement: Figure S1. — Distribution of chitinase gain and loss among fungal lineages. Phylogenetic relationships among the fungal species used in the current study are shown, including divergence dates in millions of years. Circled numbers represent total number of chitinase genes in extant species and estimates of total number of chitinase genes for ancestral species. Boxed taxon names indicates a significant (P-values ≤ 0.05 or Likelihood ratios ≥ 50) expansion (+), or a significant contraction (−) of the chitinase gene family size. [file ebo-2010-001f7.tif]

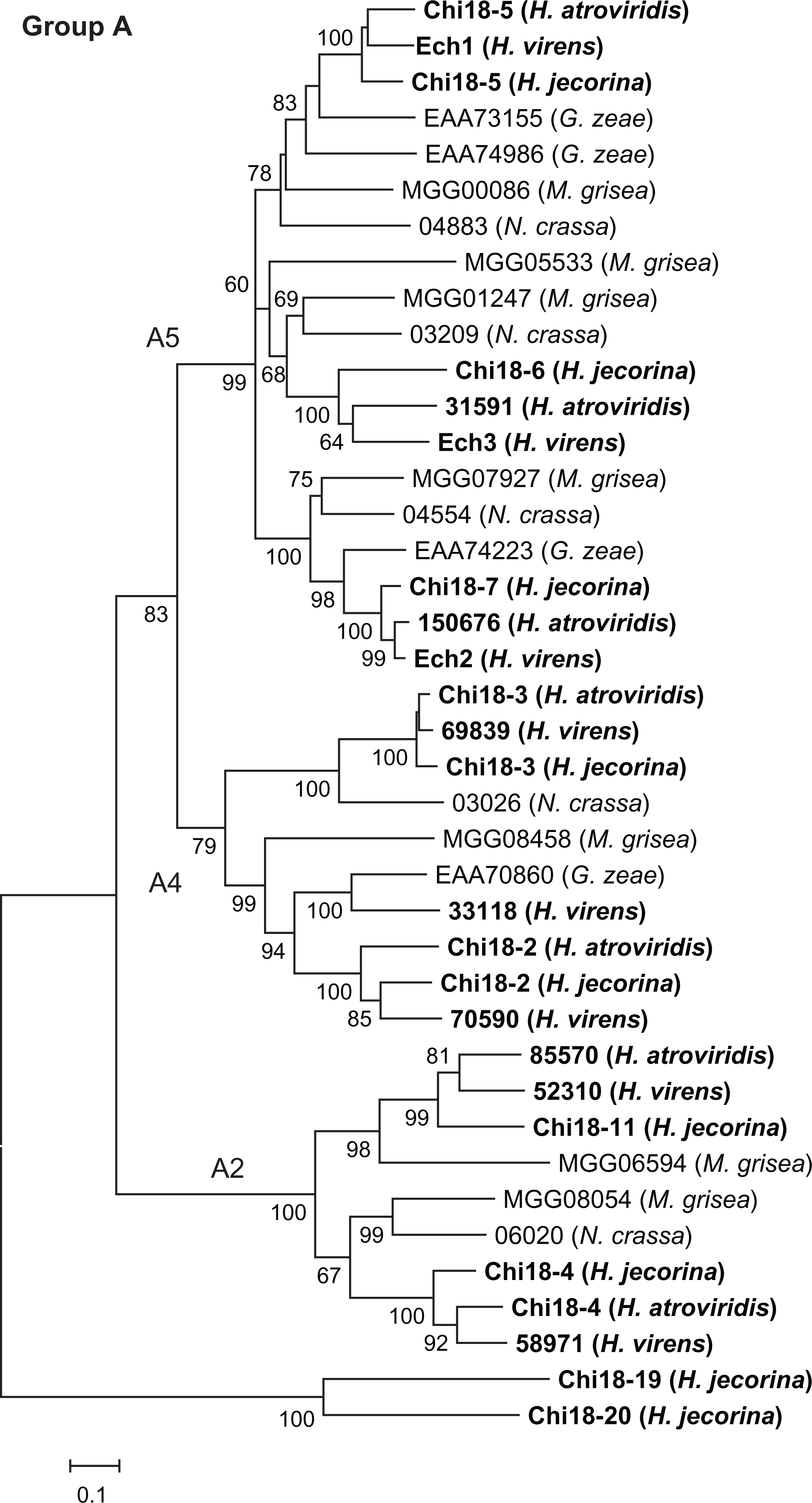

Supplement: Figure S2 Group A. — Phylogeny of group A Trichoderma chitinases. Analysis was performed using neighbour-joining implemented in MEGA version 4 with the Poisson correction of substitution rates and complete deletion of missing data, based on a Clustal W alignment of chitinase catalytic domain amino acid sequences. Branch support values (bootstrap proportions ≥ 60) are associated with nodes. The bar marker indicates numbers of amino acid substitutions. Protein identifiers include protein name (if available) or protein ID nos. from the respective genome projects. Subgroup names are indicated. [file ebo-2010-001f8.tif]

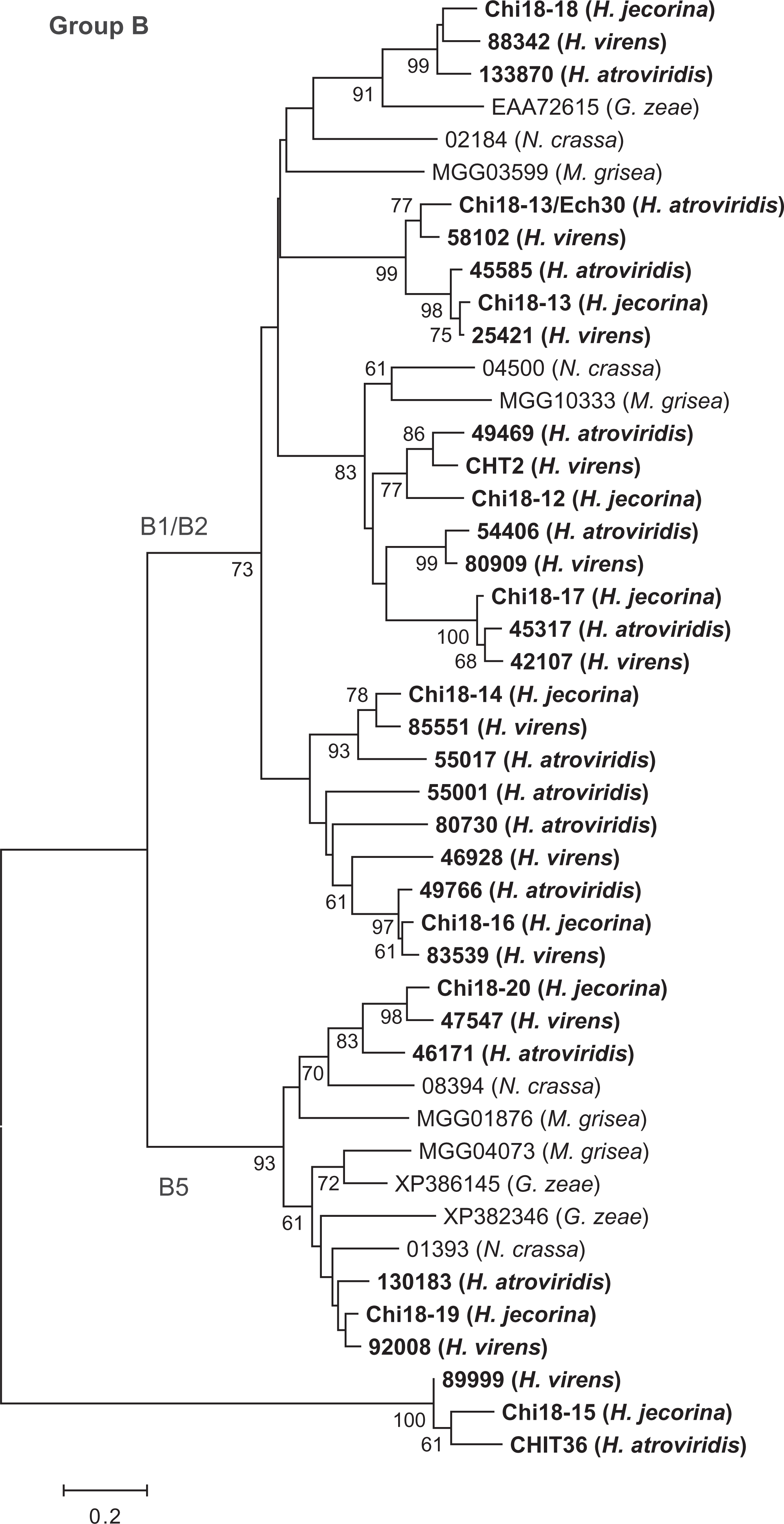

Supplement: Figure S2 Group B. — Phylogeny of group B Trichoderma chitinases. Analysis was performed using neighbour-joining implemented in MEGA version 4 with the Poisson correction of substitution rates and complete deletion of missing data, based on a Clustal W alignment of chitinase catalytic domain amino acid sequences. Branch support values (bootstrap proportions ≥ 60) are associated with nodes. The bar marker indicates numbers of amino acid substitutions. Protein identifiers include protein name (if available) or protein ID nos. from the respective genome projects. Group names are indicated. [file ebo-2010-001f9.tif]

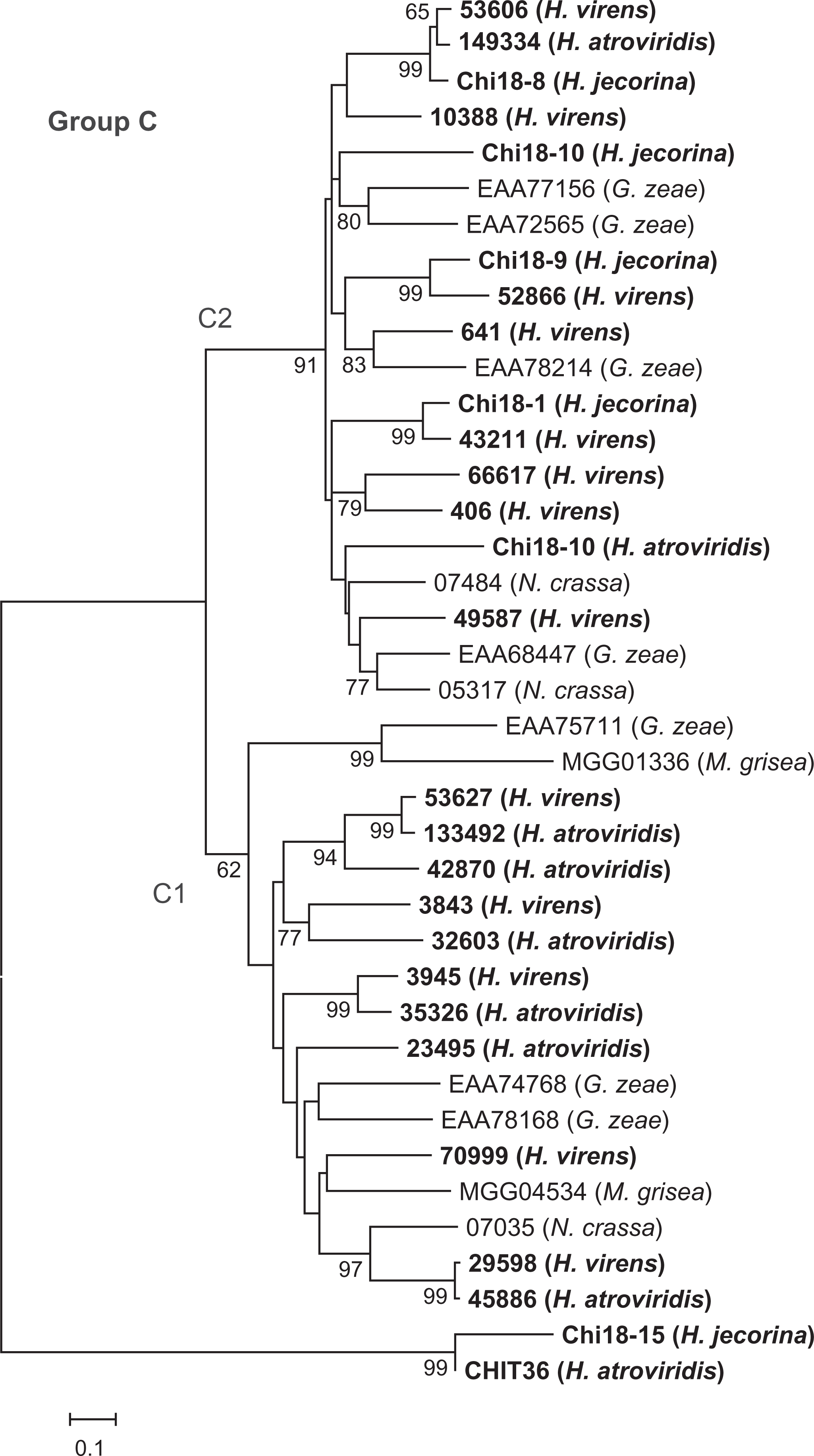

Supplement: Figure S2 Group C. — Phylogeny of group C Trichoderma chitinases. Analysis was performed using neighbour-joining implemented in MEGA version 4 with the Poisson correction of substitution rates and complete deletion of missing data, based on a Clustal W alignment of chitinase catalytic domain amino acid sequences. Branch support values (bootstrap proportions ≥ 60) are associated with nodes. The bar marker indicates numbers of amino acid substitutions. Protein identifiers include protein name (if available) or protein ID nos. from the respective genome projects. Group names are indicated. [file ebo-2010-001f10.tif]

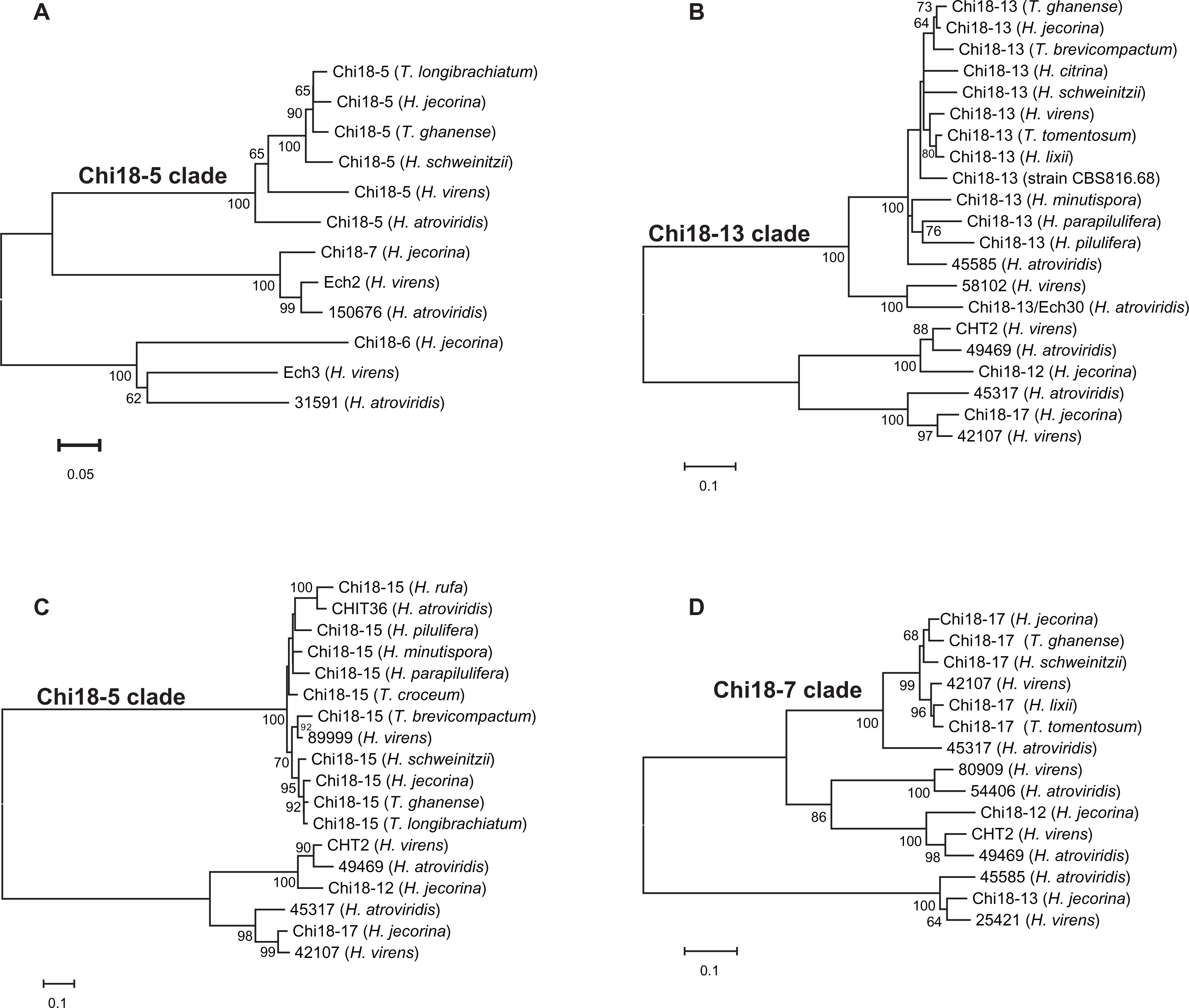

Supplement: Figure S3. — Trichoderma chitinase gene phylogenies. Analyses were performed on chitinases (A) chi18-5, (B) chi18-13, (C) chi18-15 and (D) chi18-17 using neighbour-joining implemented in MEGA version 4 with the Poisson correction of substitution rates and complete deletion of missing data, based on a Clustal W alignment of chitinase amino acid sequences. Branch support values (bootstrap proportions ≥ 60) are associated with nodes. The bar marker indicates numbers of amino acid substitutions. Protein identifiers include protein name (if available) or protein ID nos. from the respective genome projects. [file ebo-2010-001f11.tif]
